# Dipeptidyl peptidase 4 inhibitors as novel agents in improving survival in diabetic patients with colorectal cancer and lung cancer: A Surveillance Epidemiology and Endpoint Research Medicare study

**DOI:** 10.1002/cam4.2278

**Published:** 2019-05-23

**Authors:** Rohit Bishnoi, Young‐Rock Hong, Chintan Shah, Azka Ali, William P. Skelton, Jinhai Huo, Nam H. Dang, Long H. Dang

**Affiliations:** ^1^ Division of Hematology and Oncology, Department of Medicine University of Florida Gainesville FL; ^2^ Department of Health Services Research, Management and Policy, College of Public Health and Health Professions University of Florida Gainesville FL

**Keywords:** CD26, colorectal cancer, DPP4 inhibitors, lung cancer, SEER‐Medicare

## Abstract

**Background:**

Dipeptidyl peptidase 4 (DPP4) is a cell surface protein that can act as a tumor suppressor or activator, depending upon the level of expression and interaction with the microenvironment and chemokines. DPP4 inhibitors are used to treat diabetes.

**Methods:**

We conducted this Surveillance Epidemiology and Endpoint Research‐Medicare database study to evaluate the role of DPP4 inhibitors on the overall survival (OS) of diabetic patients diagnosed with colorectal (CRC) and lung cancers.

**Results:**

Diabetic patients with CRC or lung cancer who were treated with DPP4 inhibitors exhibited a statistically significant survival advantage (hazard ratio [HR] of 0.89; CI: 0.82‐0.97, *P* = 0.007) that remained significant after controlling for all other confounders. When DPP4 inhibitors were used in combination of metformin which is known to suppress cancer, the survival advantage was even more pronounced (HR of 0.83; CI: 0.77‐0.90, *P* < 0.0001). Data were then analyzed separately for two cancer types. In the CRC‐only cohort, the use of DPP4 inhibitors alone had a positive trend but did not meet statistically significant threshold (HR of 0.87; CI: 0.75‐1.00, *P* = 0.055), while the combined use of DPP4 inhibitors and metformin was associated with statistically significant survival advantage (HR of 0.77; CI: 0.67‐0.89, *P* = 0.003). Similarly, for the lung cancer cohort, use of DPP4 alone was not found to be statistically significant (HR of 0.93; CI: 0.83‐1.03, *P* = 0.153), whereas lung cancer patients treated with the combination of DPP4 inhibitors and metformin showed statistically significant survival advantage (HR of 0.88; CI: 0.80‐0.97, *P* = 0.010).

**Conclusions:**

DPP4 inhibition in CRC and lung cancer is associated with improved OS, which possibly may be due to the effect of DPP4 inhibition on immunoregulation of cancer.

## 1 INTRODUCTION

Dipeptidyl peptidase 4 (DPP4) inhibitors, also known as gliptins, are a class of oral hypoglycemic drugs that block the enzyme DPP4 and can be used to treat diabetes mellitus type 2 (DM‐II). By inhibiting DPP4, these agents increase incretin levels to inhibit glucagon release and stimulate insulin release, thereby reducing serum glucose levels. The first drug in this class was sitagliptin, which was approved by the US Food and Drug Administration (FDA) in 2006 for use in DM‐II. Since then, multiple agents in this class of drugs have been approved for this indication, and the use of this class of drug is on the rise.

Apart from the use of these drugs in the management of DM‐II, the role of DPP4 inhibitors in cancer biology has been a topic of interest in many studies. DPP4, also known as cluster of differentiation 26 (CD26), is a cell membrane protein enzyme which cleaves dipeptides from various growth factors and chemokines resulting in their enhanced degradation.^1^ DPP4/CD26 is widely expressed on different tissues as well as is present in serum and other body fluids. It plays an important role in tumor biology by acting as a tumor suppressor or activator depending upon the level of expression and its interaction with the microenvironment and selected chemokines.^1^, ^2^, ^3^ In animal models, DPP4/CD26 expression has been shown to be of prognostic value and is a potential therapeutic target in various malignancies.^4^, ^5^, ^6^, ^7^ Of note is that the first phase I clinical trial involving CD26‐expressing cancers with an anti‐CD26 monoclonal antibody was recently completed and reported prolonged disease stabilization in patients with mesothelioma with good drug tolerance.^8^

Barreira da Silva et al^9^ showed that in mice models with melanoma, DPP4 inhibition preserved the active form of chemokine CXCL10 which recruits T cells in tumor parenchyma. Their study also provided evidence that the use of a DPP4 inhibitor in combination with a programmed cell death protein 1 inhibitor and cytotoxic T lymphocyte‐associated antigen‐4 inhibitor enhances antitumor response to immunotherapy regimens. Similarly, Pereira et al showed that in mice models with melanoma, treatment with metformin or sitagliptin showed a significant reduction in the number of metastatic lung nodules. Importantly, the combination of metformin with sitagliptin showed a greater reduction in the number of metastatic lung nodules than treatment with metformin or sitagliptin alone.^10^ In the mouse xenograft model with papillary thyroid cancer, sitagliptin use was associated with reduced tumor growth, with the transforming growth factor‐β signaling pathway being potentially involved.^5^ In contradiction to these findings, Wang et al^11^ demonstrated in an in‐vivo study that use of DPP4 inhibitors increased the risk of metastasis in colon, hepatic, lung, ovary, and melanoma cell lines.

Due to these in‐vivo studies showing that DPP4/CD26 inhibition can either deter or facilitate tumor progression, we previously conducted a multi‐institutional retrospective study involving patients with advanced airway and colorectal cancers (CRCs) who were being treated for diabetes with DPP4 inhibitors. Our study, which to our knowledge was the first study evaluating the role of DPP4 inhibition on cancers in human subjects, found statistically significant benefit in progression‐free survival and a positive trend in overall survival (OS); however, this benefit in OS did not reach the level of statistical significance, likely due to the relatively small number of subjects included in the study.^12^ As a follow‐up and to further clarify the role of DPP4 inhibitors in human malignancies, we conducted a national database study in CRC and lung cancer.

## 2 MATERIALS AND METHODS

### 2.1 Databases

We utilized the Surveillance Epidemiology and Endpoint Research (SEER)‐Medicare database for our study. The SEER program, supported by the National Cancer Institute, contains cancer patients' demographic and tumor characteristics for approximately 34% of the US population. The Medicare database, maintained by the Centers for Medicare and Medicaid Services, contains health care claims and payment information, for over 97% of the US population aged 65 years or older. We used this linked SEER and Medicare databases (SEER‐Medicare), which capture detailed treatment information after cancer diagnosis from the Medicare insurance program along with individual patient level demographic and survival data from the SEER cancer registry program.

### 2.2 Study population

**Figure 1:**
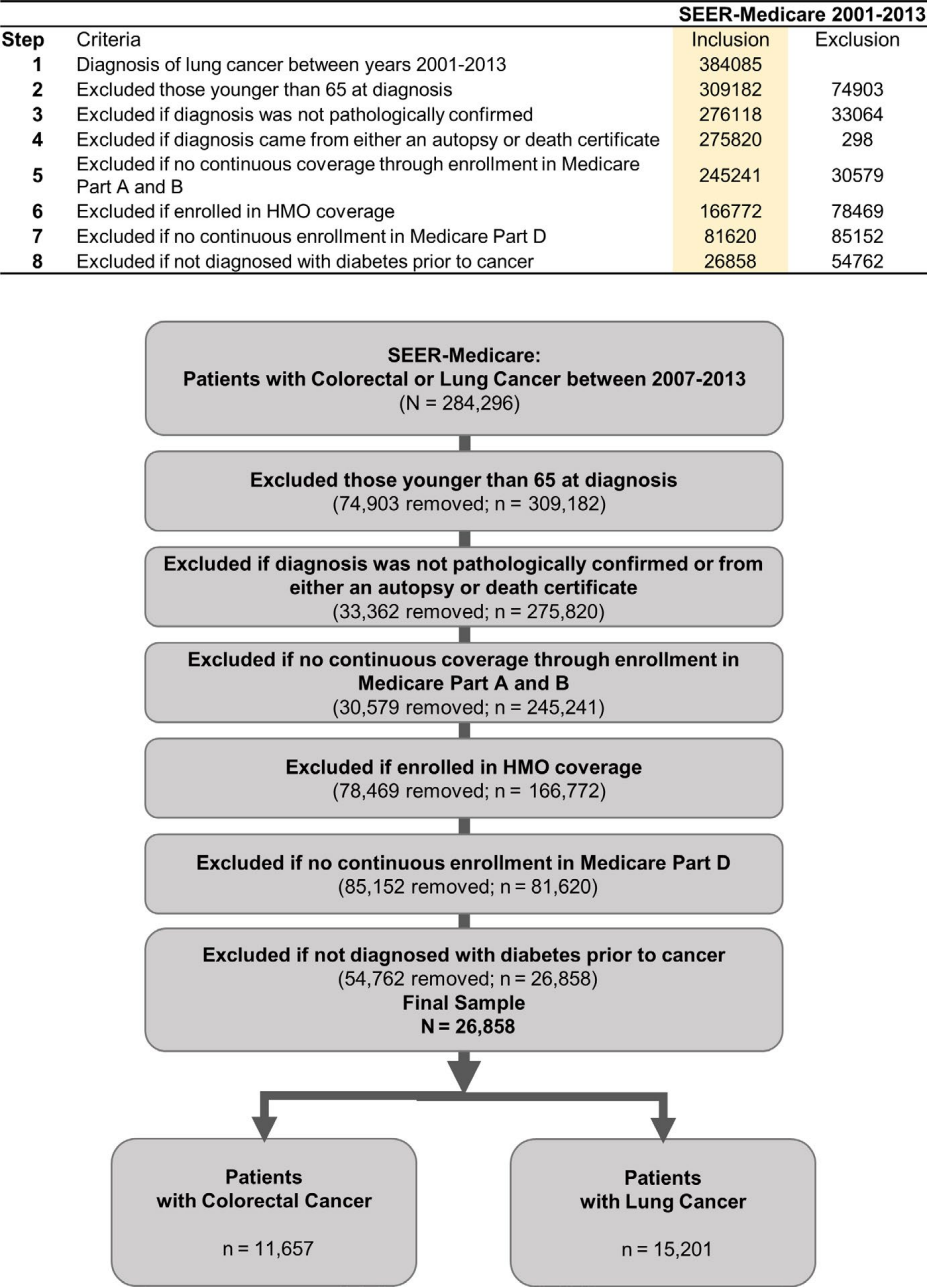
This shows the criteria and the flowchart used to identify study cohort

We used International Classification of Diseases for Oncology, third edition (ICD‐O‐3) codes to identify patients who were diagnosed with colorectal and lung cancer between 2001 and 2013 from SEER 18.^13^ We excluded patients in whom cancer diagnosis was from autopsy or death certificates or without pathological confirmation. The study samples were restricted to those with continuous Medicare Part A and Part B insurance coverage and no HMO coverage 12 months before and 12 months after a cancer diagnosis or until death. Cohort was then selected for patients who were diagnosed with diabetes prior to the diagnosis of cancer. Please refer to Figure 1 for details of cohort selection.

### 2.3 Dependent and independent variables

Use of DPP4 inhibitors following cancer diagnosis was identified using a generic name and National Drug Codes in SEER‐Medicare Part D file. DPP4 inhibitors included in the study were: alogliptin, linagliptin, saxagliptin, sitagliptin, and vildagliptin. We also used the same approach to identify the use of metformin. Patient demographic characteristics included the year of diagnosis, age, sex, race/ethnicity, marital status, census tract poverty rate, census region, urban/rural status. Patient's clinical information included cancer type, cancer stage, receipt of cancer treatment (such as surgery, chemotherapy, and radiation therapy), and comorbidity using Charlson comorbidity Index.^14^, ^15^ This index is comprised of 14 different conditions, which include cardiac, renal, liver, neurologic, and dementia among others. The type of cancer treatment received within 1 year of cancer diagnosis was identified using both ICD (ninth revision) procedure codes and level II Healthcare Common Procedure Coding System: Current Procedural Terminology codes in the Medicare claims. We used the modified algorithm by Klabunde et al^14^, ^15^ to calculate the Charlson Comorbidity Index.

### 2.4 Study cohorts and statistical analysis

Metformin is another antidiabetic drug, which is widely prescribed and is probably the most common drug used for the treatment of DM‐II. It is also is well‐established fact that metformin has a role in tumor suppression and improving OS in multiple cancers, including colorectal and lung cancer.^16^, ^17^, ^18^, ^19^ To avoid confounding by metformin use, we divided the study cohort into four groups for survival analysis. We classified patients into four groups with respect to use of DPP4 inhibitors and metformin: (a) not on either medication (not DPP4 inhibitors nor metformin; reference group), (b) on metformin only, (c) on DPP4 inhibitors only, and (d) on DPP4 inhibitors with metformin (combination group). We used the Cox Proportional Hazards survival model to assess the outcome of the four groups, controlling for patient's demographic and clinical characteristics. Reference group in our study included diabetic patients with CRC or lung cancer and not on metformin or DPP4 inhibitors.

Bivariate analyses compared baseline characteristics between DPP4 inhibitors users and nonusers using Pearson chi‐square tests. The survival time was defined as from the date of cancer diagnosis until date of death or loss of follow‐up. The criterion for statistical significance was a *P* < 0.05. All statistical analyses were conducted using SAS software (version 9.4; SAS Institute, Cary, NC, USA). The University of Florida institutional review board approved this study.

## 3 RESULTS

A total of 26 858 numbers of patients were found to have the diagnosis of diabetes and developed either CRC or Lung cancer. Of 26 858 patients, 11 657 had CRC while 15 201 had lung cancer. Of these, 1786 patients were on DPP4 inhibitors for DM‐II. Table 1 shows general demographic information about the cohort. As seen in this table, use of DPP4 inhibitors has been increasing since the time of FDA approval in 2006 (2.6% in 2007 to 24% in 2013). Of these 1786 patients, 1011 were on DPP4 inhibitors in combination metformin, and 775 were only on DPP4 inhibitors.

**Table 1:** General demographics of the cohort by DPP4 inhibition

Characteristic	Total	DPP4				*P*‐value
		No	%	Yes	%	
Demographics						
Year of diagnosis						<0.0001
2007	2261	2214	8.8	47	2.6	
2008	4003	3858	15.4	145	8.1	
2009	4128	3962	15.8	166	9.3	
2010	3953	3718	14.8	235	13.2	
2011	3981	3651	14.6	330	18.5	
2012	4236	3801	15.2	435	24.4	
2013	4296	3868	15.4	428	24.0	
Age group						0.201
65‐69	5301	4938	19.7	363	20.3	
70‐74	6844	6367	25.4	477	26.7	
75‐79	6156	5739	22.9	417	23.3	
80+	8557	8028	32.0	529	29.6	
Sex						0.018
Male	12 451	11 575	46.2	876	49.0	
Female	14 407	13 497	53.8	910	51.0	
Race/Ethnicity						<0.0001
Non‐Hispanic White	19 012	17 812	71	1200	67.2	
Non‐Hispanic Black	3132	2975	11.9	157	8.8	
Hispanic	2462	2263	9.0	199	11.1	
Others	2252	2022	8.1	230	12.9	
Marital status						0.0006
Single	5930	5586	22.3	344	19.3	
Married	11 379	10 551	42.1	828	46.4	
Other (Sep/Div/Wid/Unknown)	9549	8935	35.6	614	34.4	
Census poverty						0.058
0% to <5% poverty	4680	4335	17.3	345	19.3	
5% to <10%	6131	5736	22.9	395	22.1	
10%‐20%	8098	7584	30.2	514	28.8	
20%‐100%	7444	6956	27.7	488	27.3	
Unknown	505	461	1.8	44	2.5	
Census region						<0.0001
West	9818	9072	36.2	746	41.8	
Northeast	6280	5841	23.3	439	24.6	
Midwest	3084	2952	11.8	132	7.4	
South	7676	7207	28.7	469	26.3	
Rural/urban status						0.085
Urban area	26 147	24 397	97.3	1750	98.0	
Rural area	711	675	2.7	36	2.0	
Charlson Comorbid Index						<0.0001
0	2471	2436	9.7	35	2.0	
1	8139	7623	30.4	516	28.9	
2	6381	5916	23.6	465	26.0	
3+	9867	9097	36.3	770	43.1	
Cancer type						0.105
Colorectal cancer	11 657	10 849	43.3	808	45.2	
Lung cancer	15 201	14 223	56.7	978	54.8	
Stage						0.221
I	5947	5543	22.1	404	22.6	
II	3444	3219	12.8	225	12.6	
III	6352	5927	23.6	425	23.8	
IV	8156	7592	30.3	564	31.6	
Unknown	2959	2791	11.1	168	9.4	
Surgery						0.355
No	19 219	17 958	71.6	1261	70.6	
Yes	7639	7114	28.4	525	29.4	
Chemotherapy						0.0791
No	21 470	20 071	80.1	1399	78.3	
Yes	5388	5001	19.9	387	21.7	
Radiotherapy						0.566
No	24 472	22 838	91.1	1634	91.5	
Yes	2386	2234	8.9	152	8.5	

Abbreviation: DPP4, dipeptidyl peptidase 4.

Our analysis found that use of DPP4 inhibitors by itself or in combination with metformin improved OS, which was statistically significant. Taking the entire study cohorts of CRC and lung cancers together, patients using DPP4 inhibitors only (n = 775) had hazard ratio (HR) of 0.89 (95% CI: 0.82‐0.97, *P* = 0.007) while the patients with combined use of DPP4 inhibitors and metformin (n = 1011) showed HR 0.83 (95% CI: 0.77‐0.90, *P* < 0.0001).

The analysis was further performed for CRC and lung cancer cohorts independently. In CRC cohort, patients using DPP4 inhibitor only (n = 356) had HR of 0.87 (95% CI: 0.75‐1.00, *P* = 0.055) while patients with combined use of DPP4 inhibitors and metformin (n = 452) showed HR of 0.77 (95% CI: 0.67‐0.89, *P* = 0.003). In lung cancer patients, DPP4 inhibitor only group (n = 419) showed HR of 0.93 (95% CI: 0.83‐1.03, *P* = 0.153) while the group with combined use of DPP4 inhibitors and metformin (n = 559) showed HR of 0.88 (95% CI: 0.80‐0.97, *P* = 0.010).

**Figure 2:**
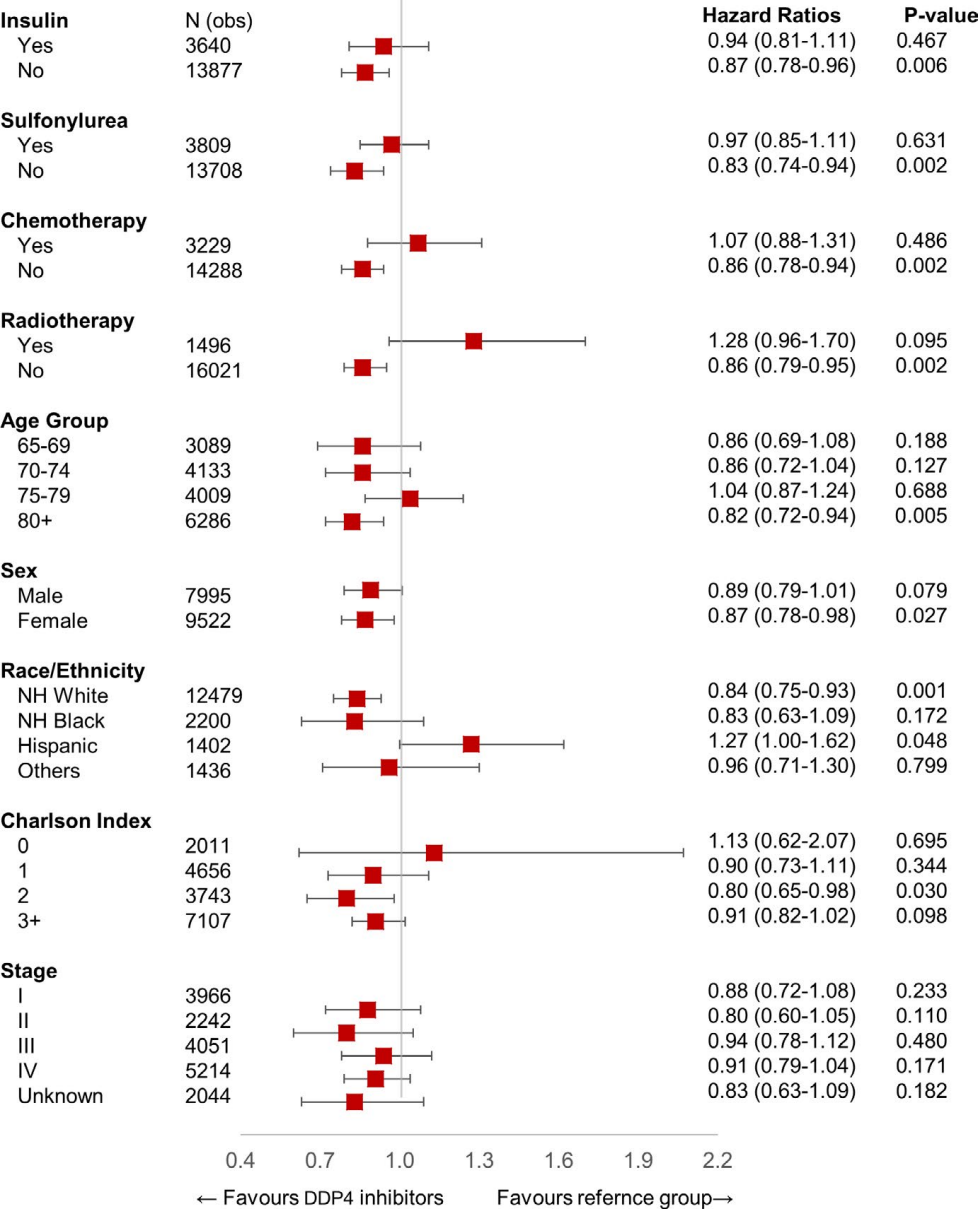
HR of patients using DPP4 inhibitor only (n = 775) in interaction with other variables in the study population after excluding patients treated with metformin (n = 17 517). DPP4, dipeptidyl peptidase 4; HR, hazard ratio

**Figure 3:**
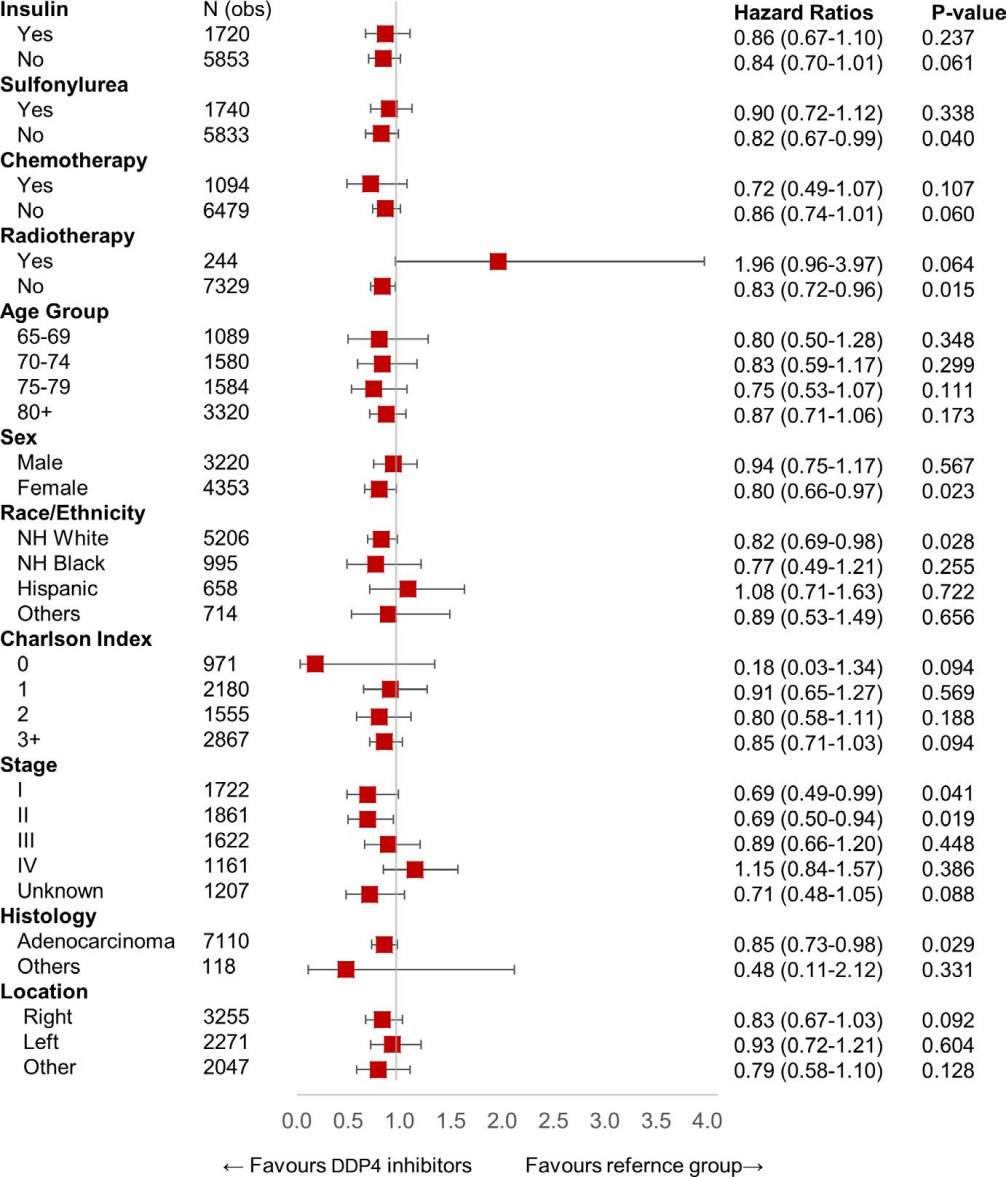
HR of patients using DPP4 inhibitor only (n = 356) in interaction with other variables in CRC cohort (n = 7573). DPP4, dipeptidyl peptidase 4; HR, hazard ratio

**Figure 4:**
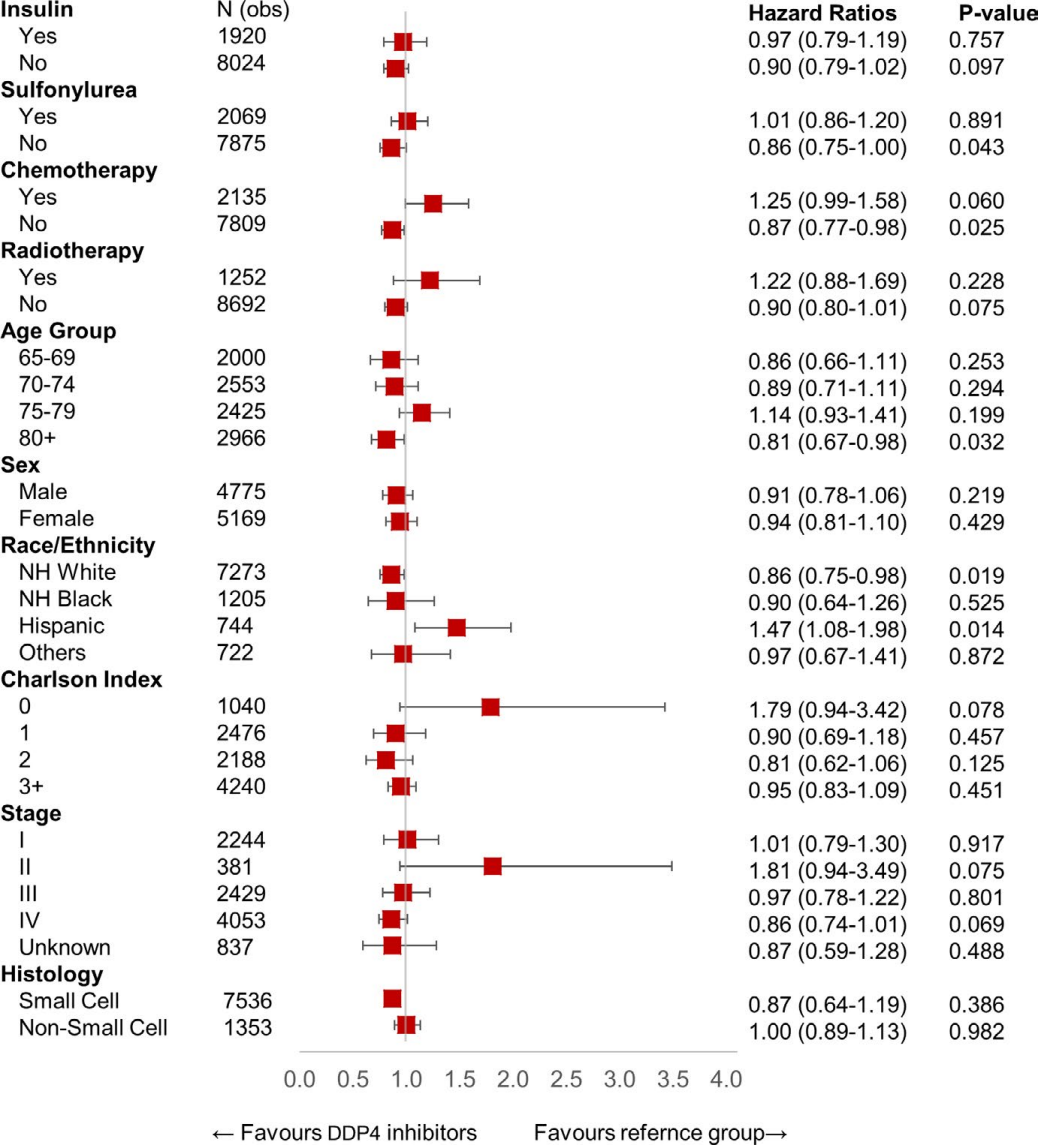
HR of patients using DPP4 inhibitor only (n = 419) in interaction with other variables in Lung cancer cohort (n = 9944). DPP4, dipeptidyl peptidase 4; HR, hazard ratio

After primary analysis, we performed further analysis to identify any other demographic, treatment, or cancer‐related variable that was significant in patients on DPP4 inhibitors. In this analysis, we excluded patients who were on metformin to remove this confounding variable. Figure 2 represents a combined cohort of CRC plus Lung cancer patients (n = 17 517), Figure 3 shows analysis for CRC patients (n = 7573), and Figure 4 shows analysis for lung cancer patients (n = 9944). In this analysis, we saw a persistent and significant HR for females and White people in the combined cohort of lung cancer and CRC patients, as well as in lung cancer patients only and CRC patients only cohorts. We did find other significant variables, but these findings were not consistent throughout the groups. Please see the attached figures for other variables.

## 4 DISCUSSION

Our study included two groups of cancers, CRC and lung cancers, which have a different clinical course. We included the same group of cancers as we included in our initial retrospective study.^12^ With a larger sample size, our present study showed statistically significant HR in the combined cohort of CRC and lung cancer patients. Results showed that DPP4 inhibitor use was associated with an improved OS with a statistically significant HR. This effect was seen with DPP4 inhibitors use, either by itself (HR 0.89) or in combination with metformin (HR 0.83). On analysis of individual cohorts of CRC and lung cancers, results were still statistically significant for the combined use of DPP4 inhibitors and metformin in CRC (HR 0.77) as well as for lung cancer (HR 0.88).

However, the results did not meet statistically significant threshold for isolated use of DPP4 inhibitors in separate cohorts of lung and CRC, which we believe is due to smaller sample size. Results were still very encouraging for the CRC cohort with patients treated with DPP4 inhibitors showing a positive trend in improved survival but not meeting statistically significant threshold with HR of 0.87 (95% CI: 0.75‐1.00, *P* = 0.055). In patients with lung cancer, similar analysis showed HR of 0.93 (95% CI 0.83‐1.03, *P* = 0.153), which was not significant. This difference again can be related to the smaller sample size as well as the different clinical course of these two different diseases. We performed detailed analysis for different variables and their interaction with DPP4 inhibitor use as compared to the reference group. Although we did find some subgroups that showed significant benefit from DPP4 inhibitor use such as White race and female gender, the clinical significance of these findings and the underlying mechanism are unclear. It will be interesting to see if these findings can be replicated with larger sample size or prospective trials.

**Figure 5:**
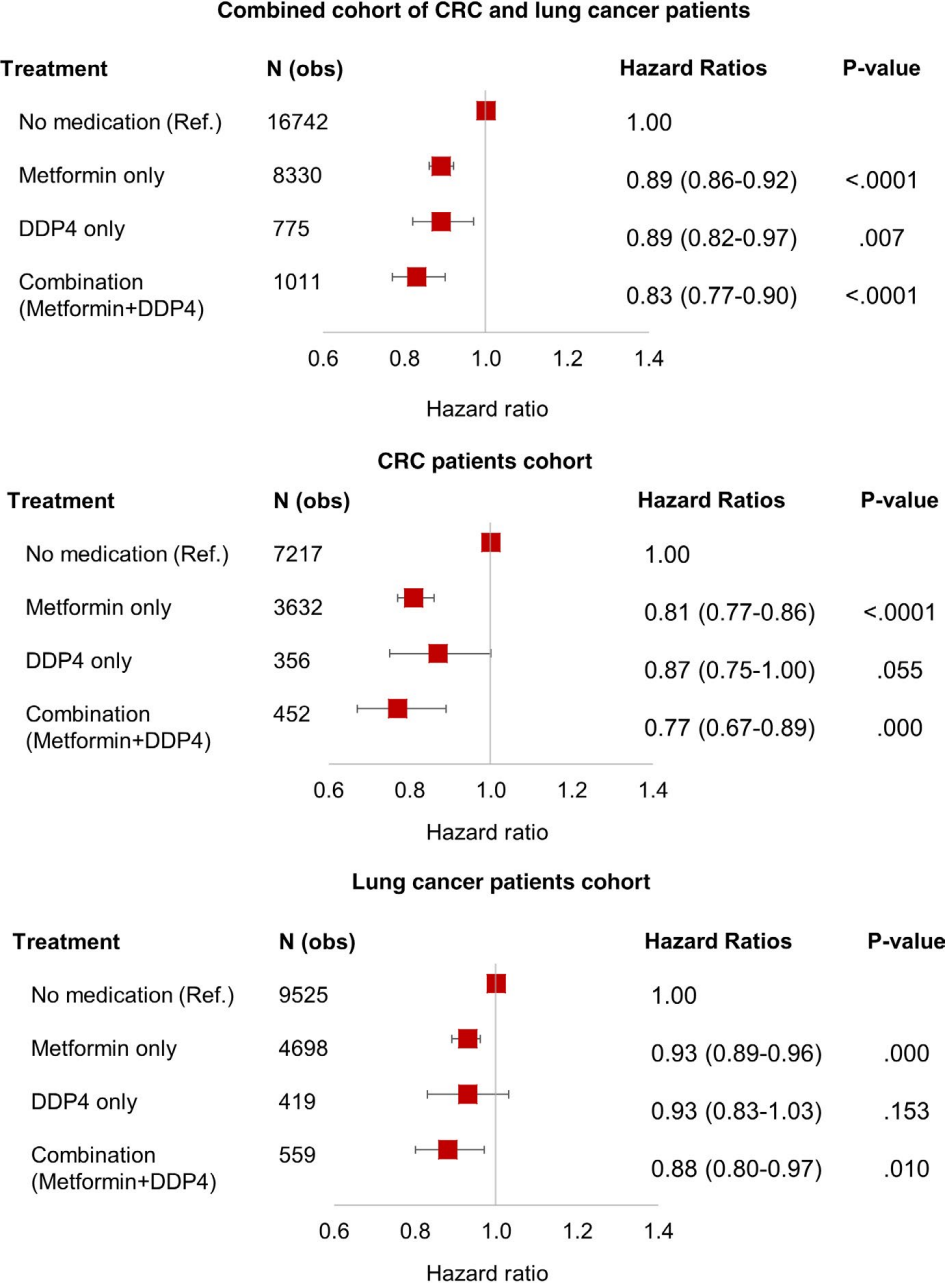
This shows the multivariable analysis of combined cohort, CRC, and lung cancer patients. CRC, colorectal cancer

Metformin, which is known to have an adjunctive protective role in malignancy, was again found to be statistically significant in a combined cohort of CRC and lung, as well as separately in each CRC and lung cancer cohort. While the role of metformin is well‐established, it was interesting to find that the combined use of DPP4 inhibitors with metformin had a lower HR as compared to metformin or DPP4 inhibitors alone (Figure 5). These findings are consistent in our analysis in a combined cohort of CRC and lung cancer as well as independent cohorts of CRC or lung cancer. Although there is no direct comparison, the comparative improvement of HR for a combination of DPP4 inhibitors and metformin as compared to either drug alone is encouraging and suggests an additive effect on OS of the combination. This finding was also noticed in the in‐vivo study by Pereira et al.^10^ Our study is based on SEER‐Medicare data until 2013 prior to the use of immunotherapy drugs for cancer treatment. It will be interesting to see how DPP4 inhibitors interplay with immunotherapy drugs as suggested by Barreira da Silva et al.^9^

As we mentioned before, various preclinical or in‐vivo studies have shown the role of DPP4/CD26 in either suppressing or activating tumor cells. We believe that DPP4/CD26 exerts its effects on tumor cells by interacting with immune cells through chemokines, but exact mechanisms are still unknown. Immune response against tumor cells depends on the infiltration of the tumor microenvironment by T cells, which in turn is guided by chemokines. DPP4/CD26 cleaves dipeptides from certain chemokines leading to their degradation, which limits infiltration of effector T cell into solid tumors. Thus, inhibition of DPP4 enzymatic activity by DPP4 inhibitors would limit degradation of chemokines, enhance tumor infiltration by T cells, and tumor cell killing.

### 4.1 Study limitations

SEER database includes data from 19 different geographical areas covering approximately 34% of the US population. Although this dataset includes patient population from diverse demographics and locations, it still does not include the entire US population.^20^ Therefore, both data and results might be affected by various local risk factors, including access to health care, and caution should be exerted before generalization.^21^ Also, this study is retrospective in nature that carries limitations of a retrospective study and prevent us from drawing direct causal inferences regarding the use of DPP4 inhibitors and survival outcomes. Moreover, our hypothesis of the underlying mechanism of action by enhanced immune response is applicable to any cancer or histologic type. The sample size was another significant limitation, and we believe larger sample size would have consolidated the positive trends noted in this study to statistically significant findings.

## 5 CONCLUSION

This SEER‐Medicare study further establishes the role of DPP4 inhibitors as cancer inhibitory, especially in combination with metformin. We plan to follow these findings with prospective trials.

## CONFLICT OF INTEREST

The authors declare that they have no conflict of interest.

## AUTHOR CONTRIBUTIONS

Long H. Dang, Nam H. Dang, Chintan Shah, Jinhai Huo, and Rohit Bishnoi designed the study. Jinhai Huo, Nam H. Dang, and Long H. Dang are principal investigators and mentor authors with equal contribution. Rohit Bishnoi and Young‐Rock Hong were major contributor in writing manuscript. Long H. Dang, Nam H. Dang, Chintan Shah, Jinhai Huo, Azka Ali, and William Paul Skelton IV contributed in drafting, editing, and revising the manuscript. Jinhai Huo and Young‐Rock Hong provided statistical analysis. All authors read and approved the final manuscript.

## ETHICS APPROVAL AND CONSENT TO PARTICIPATE

The Institution Review Board (IRB) at the University of Florida, Gainesville FL, approved this study and all standard ethical guidelines were followed. Full waiver of informed consent was obtained.

## CONSENT TO PUBLICATION

Not applicable. The manuscript does not contain any individual's data.

## Data Availability

The datasets and study analysis details are available from the corresponding author on reasonable request.
